# Anti-cancer activity of Psoralea fructus through the downregulation of cyclin D1 and CDK4 in human colorectal cancer cells

**DOI:** 10.1186/s12906-016-1364-x

**Published:** 2016-09-26

**Authors:** Gwang Hun Park, Ji Ho Sung, Hun Min Song, Jin Boo Jeong

**Affiliations:** Department of Medicinal Plant Resources, Andong National University, Andong, 36729 Republic of Korea

**Keywords:** Anticancer, CDK4, Cyclin D1, Psoralea Fructus, *Psoralea corylifolia*

## Abstract

**Background:**

Psoralea Fructus (PF), the dried and ripe fruit of *Psoralea corylifolia* exhibits an anti-cancer activity. However, the molecular mechanisms by which PF inhibits the proliferation of cancer cells have not been elucidated in detail. Cyclin D1 and CDK4 are important regulatory proteins in cell growth and are overexpressed in many cancer cells. In this study, we investigated the molecular mechanism of PF on the downregulation of cyclin D1 and CDK4 level.

**Methods:**

Cell growth was evaluated by MTT assay. The effect of PF on cyclin D1 and CDK4 expression was evaluated by Western blot or RT-PCR.

**Results:**

PF suppressed the proliferation of human colorectal cancer cell lines such as HCT116 (IC_50_: 45.3 ± 1.2 μg/ml), SW480 (IC_50_: 37.9 ± 1.6 μg/ml), LoVo (IC_50_: 23.3 ± 1.9 μg/ml μg/ml) HT-29 (IC_50_ value: 40.7 ± 1.5 μg/ml). PF induced decrease in the protein expression of cyclin D1 and CDK4. However, the mRNA expression of cyclin D1 and CDK4 did not be changed by PF; rather it suppressed the expression of cyclin D1 and CDK4 via the proteasomal degradation. In cyclin D1 degradation, we found that T286 of cyclin D1 play a pivotal role in PF-mediated cyclin D1 degradation. Subsequent experiments with several kinase inhibitors suggest that PF-mediated degradation of cyclin D1 and CDK4 is dependent on ERK1/2 and/or GSK3β.

**Conclusions:**

Our results suggest that PF has potential to be a candidate for the development of chemoprevention or therapeutic agents for human colorectal cancer.

## Background

Medicinal herbals have gained increasing attention for their effectiveness and relatively minor side effects [1]. Psoralea Fructus (PF), the dried and ripe fruit of *Psoralea corylifolia* known as “*Bo-Gol-Zhee*” in Korea has been used as traditional medicine in Asian [2]. PF has been demonstrated to exert unique effectiveness against infectious disease, inflammatory disorder, tumor and depression [3–5]. Many active components have been identified in PF, and most of them belong to coumarins (psoralen, isopsoralen, and psoralidin) and flavonoids (bavachin, isobavachalcone and neobavaisoflavone) [6]. These compounds show anti-oxidative, anti-tumor, anti-bacterial, and protective effects on cutaneous complaints, impotence, and hepatic injury [7]. In anticancer activity, PF has been reported to induce the cell growth arrest and apoptosis in human oral carcinoma lines and erythroleukemia cells [5]. However, the potential mechanism by which PF induces the cell growth arrest has remained unknown.

Cyclin D1 as an oncogenic protein facilitates cell cycle progression via several transcriptional factor by an active complex with cyclin-dependent kinase 4/6 (CDK4/6) [8]. Overexpression of cyclin D1 found in various cancer cells is associated with the poor prognosis of tumor and metastasis [9]. Especially, cyclin D1 overexpression has been overexpressed in 68.3 % of human colorectal cancer case and deregulation of cyclin D1 has been associated with colorectal tumorigenesis [10, 11]. Thus, it has been accepted that cyclin D1 has been an attractive chemopreventive and therapeutic target for anti-cancer development [12]. In addition, mutation or amplification of CDK4 is associated with the transition from G1 to S phase and ultimately induces cancer cell growth [13]. CDK4 has been reported to be overexpressed in breast, head and neck and lung cancer cells [13]. CDK4 overexpression has been observed in 87 % of human colorectal cancer [14]. Thus, the CDK4 inhibition offers an attractive therapeutic strategy for anti-cancer development due to the importance of CDK4 activity in regulating cell proliferation [13].

In the current study, we evaluated the effect of the ethanol extracts from PF on cyclin D1 and CDK4 suppression in the colorectal cancer cells. We found that the ethanol extracts from Psoralea fructus down-regulates cyclin D1 and CDK4 via a proteasomal-dependent pathway.

## Methods

### Materials

Cell culture media, Dulbecco’s Modified Eagle medium (DMEM)/F-12 1:1 Modified medium (DMEM/F-12) was purchased from Lonza (Walkersville, MD, USA). PD98059, SB203580, SP600125, LiCl, MG132 and 3-(4,5-dimethylthizaol-2-yl)-2,5-diphenyl tetrazolium bromide (MTT) were purchased from Sigma Aldrich (St. Louis, MO, USA). Antibodies against cyclin D1, phospho-cyclin D1 (Thr286), HA-tag, CDK4, p-ERK1/2, total-ERK1/2, p-GSK3β, total-GSK3β and β-actin were purchased from Cell Signaling (Bervely, MA, USA). All chemicals were purchased from Fisher Scientific, unless otherwise specified.

### Sample preparation

Psoralea Fructus (PF), the dried and ripe fruit of *Psoralea corylifolia* were kindly provided by the Bonghwa Alpine Medicinal Plant Experiment Station, Korea. Psoralea Fructus (voucher number: Koo001(ANH)) was formally identified by Jin Suk Koo as the professor of Andong National University, Korea. Two hundred gram of PF was extracted with l L of 70 % ethanol with shaking for 48 h. After 48 h, the ethanol-soluble fraction was filtered and concentrated to approximately 300 ml volume using a vacuum evaporator and then freeze-dried. The ethanol extracts from PF was kept in a refrigerator until use.

### Cell culture and treatment

Human colorectal cancer cell lines such as HCT116, SW480, LoVo and HT-29 were purchased from Korean Cell Line Bank (Seoul, Korea) and grown in DMEM/F-12 supplemented with 10 % fatal bovine serum (FBS), 100 U/ml penicillin and 100 μg/ml streptomycin. The cells were maintained at 37 ^o^C under a humidified atmosphere of 5 % CO_2_. The ethanol extracts from Psoralea Fructus (PF) was dissolved in dimethyl sulfoxide (DMSO) and treated to cells. DMSO was used as a vehicle and the final DMSO concentration did not exceed 0.1 % (v/v).

### Cell proliferation assay

Cell growth was measured using MTT assay system. Briefly, cells were plated onto 96-well plated and grown overnight. The cells were treated with 0, 25, 50 and 100 μg/ml of PF for 24 h. Then, the cells were incubated with 50 μl of MTT solution (1 mg/ml) for an additional 2 h. The resulting crystals were dissolved in DMSO. The formation of formazan was measured by reading absorbance at a wavelength of 570 nm.

### SDS-PAGE and Western blot

After PF treatment, cells were washed with 1 × phosphate-buffered saline (PBS), and lysed in radioimmunoprecipitation assay (RIPA) buffer (Boston Bio Products, Ashland, MA, USA) supplemented with protease inhibitor cocktail (Sigma-Aldrich) and phosphatase inhibitor cocktail (Sigma-Aldrich), and centrifuged at 15,000 × g for 10 min at 4 °C. Protein concentration was determined by the bicinchoninic acid (BCA) protein assay (Pierce, Rockford, IL, USA). The proteins were separated on SDS-PAGE and transferred to PVDF membrane (Bio-Rad Laboratories, Inc., Hercules, CA, USA). The membranes were blocked for non-specific binding with 5 % non-fat dry milk in Tris-buffered saline containing 0.05 % Tween 20 (TBS-T) for 1 h at room temperature and then incubated with specific primary antibodies in 5 % non-fat dry milk at 4 °C overnight. After three washes with TBS-T, the blots were incubated with horse radish peroxidase (HRP)-conjugated immunoglobulin G (IgG) for 1 h at room temperature and chemiluminescence was detected with ECL Western blotting substrate (Amersham Biosciences, Piscataway, NJ, USA) and visualized in Polaroid film.

### Reverse transcriptase-polymerase chain reaction (RT-PCR)

After PF treatment, total RNA was prepared using a RNeasy Mini Kit (Qiagen, Valencia, CA, USA) and total RNA (1 μg) was reverse-transcribed using a Verso cDNA Kit (Thermo Scientific, Pittsburgh, PA, USA) according to the manufacturer’s protocol for cDNA synthesis. PCR was carried out using PCR Master Mix Kit (Promega, Madison, WI, USA) with human primers for cyclin D1, CDK4 and GAPDH as followed : cyclin D1: forward 5′-aactacctggaccgcttcct-3′ and reverse 5′-ccacttgagcttgttcacca-3′, CDK4: forward 5′-atggctgccactcgatatgaaccc-3′ and reverse 5′-gtaccagagcgtaaccaccacagg-3′, GAPDH: forward 5′-acccagaagactgtggatgg-3′ and reverse 5′-ttctagacggcaggtcaggt-3′.

### Expression vectors

Wild type HA-tagged cyclin D1 and point mutation of T286A of HA-tagged cyclin D1 were provided from Addgene (Cambridge, MA, USA). Transient transfection of the vectors was performed using the PolyJet DNA transfection reagent (SignaGen Laboratories, Ijamsville, MD, USA) according to the manufacturers’ instruction.

### Statistical analysis

All the data are shown as mean ± SEM (standard error of mean). Statistical analysis was performed with one-way ANOVA followed by Dunnett’s test. Differences with *P < 0.05 were considered statistically significant.

## Results

### PF inhibits cell proliferation, and decreases the expression of cyclin D1 and CDK4

**Fig. 1:**
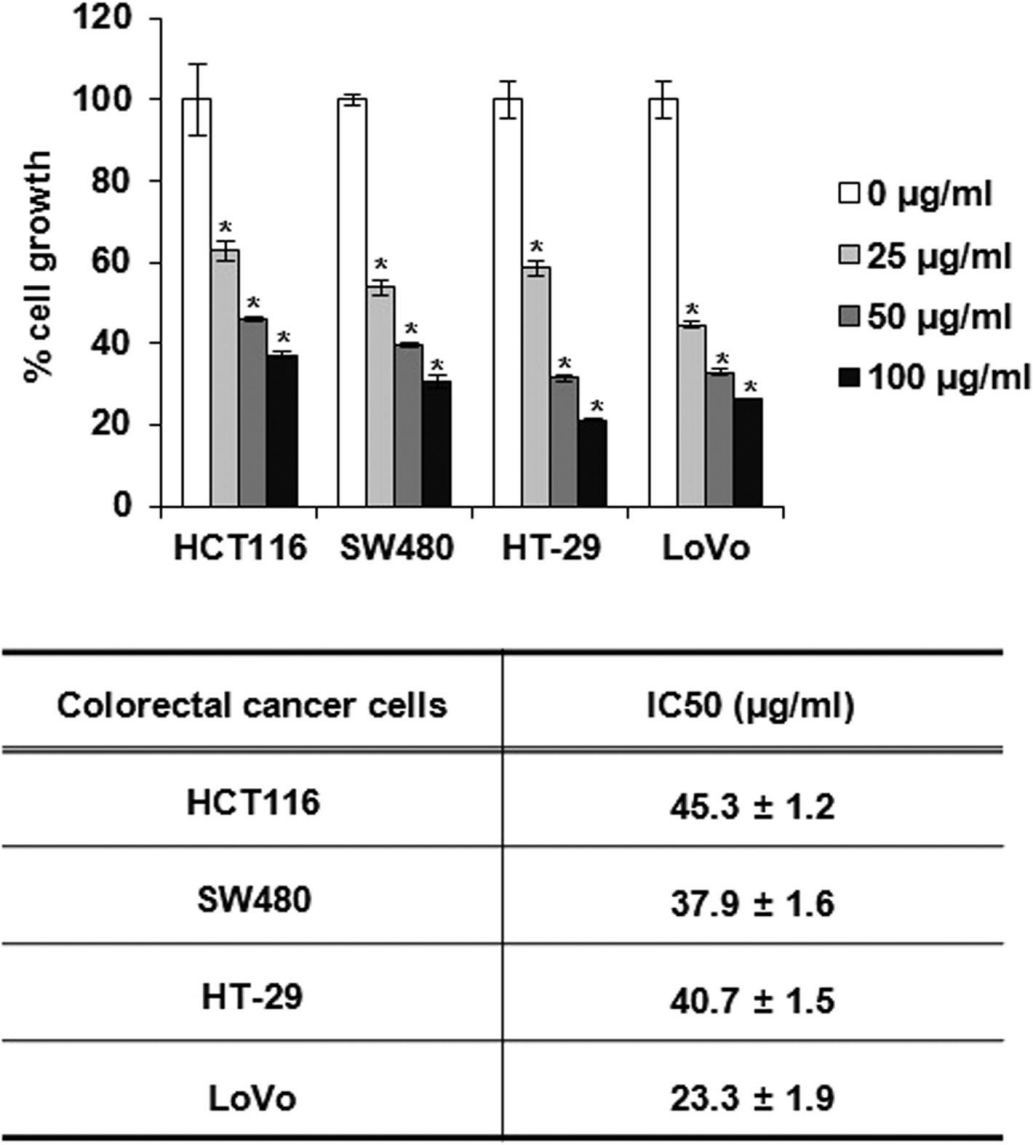
The effect on PF on the cell proliferation in human colorectal cancer cells. The cells were plated overnight and then treated with PF for 24 h. Cell proliferation was measured using MTT assay as described in Materials and methods. *P < 0.05 compared to cell without PF treatment

**Fig. 2:**
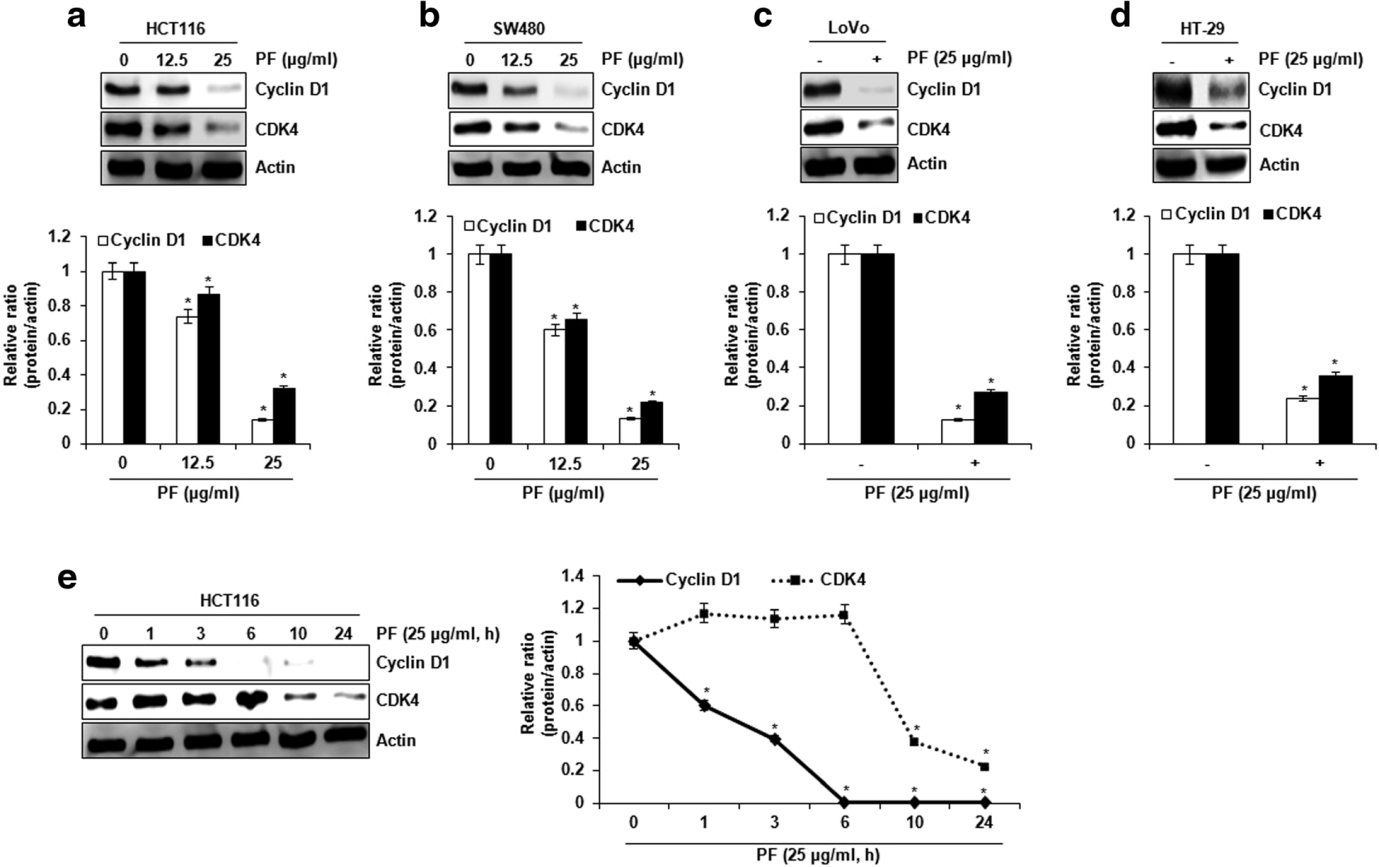
The effect on PF on the protein expression of cyclin D1 and CDK4 in human colorectal cancer cells. **a**-**d** The cells were treated with the indicated concentrations of PF for 24 h. **e** HCT116 cells treated with PF (25 μg/ml) for the indicated times. Cell lysates were subjected to SDS-PAGE and the Western blot was performed using antibodies against cyclin D1 and CDK4. Actin was used as internal control for Western blot analysis. *P < 0.05 compared to cell without PF treatment

To evaluate whether PF affects the proliferation of human colorectal cancer cells, MTT assay was performed. As shown in Fig. 1, PF treatment for 24 h suppressed the cell growth of HCT116 and SW480 cells by 38 % and 47 % at 25 μg/ml, 54 % and 61 % at 50 μg/ml, and 63 % and 70 % at 100 μg/ml, respectively. In addition, the proliferation of HT-29 and LoVo cells was inhibited by PF treatment at 42 % and 56 % at 25 μg/ml, 69 % and 68 % at 50 μg/ml, and 79 % and 74 % at 100 μg/ml, respectively. Since cell growth inhibition is related to cell cycle arrest, we investigated the expression of cyclin D1 and CDK4 involved in cell cycle progression. The cells were treated with 12.5 and 25 μg/ml for 24 h and Western blot was performed. As shown in Fig. 2a-d, the proteins of cyclin D1 and CDK4 were down-regulated by PF treatment. In time-course experiment (Fig. 2e), the protein expression of cyclin D1 started to be decreased at 1 h after PF treatment, while CDK4 expression was suppressed at 10 h after PF treatment.

### Effect of PF on the transcriptional and post-translational regulation of cyclin D1 and CDK4

**Fig. 3:**
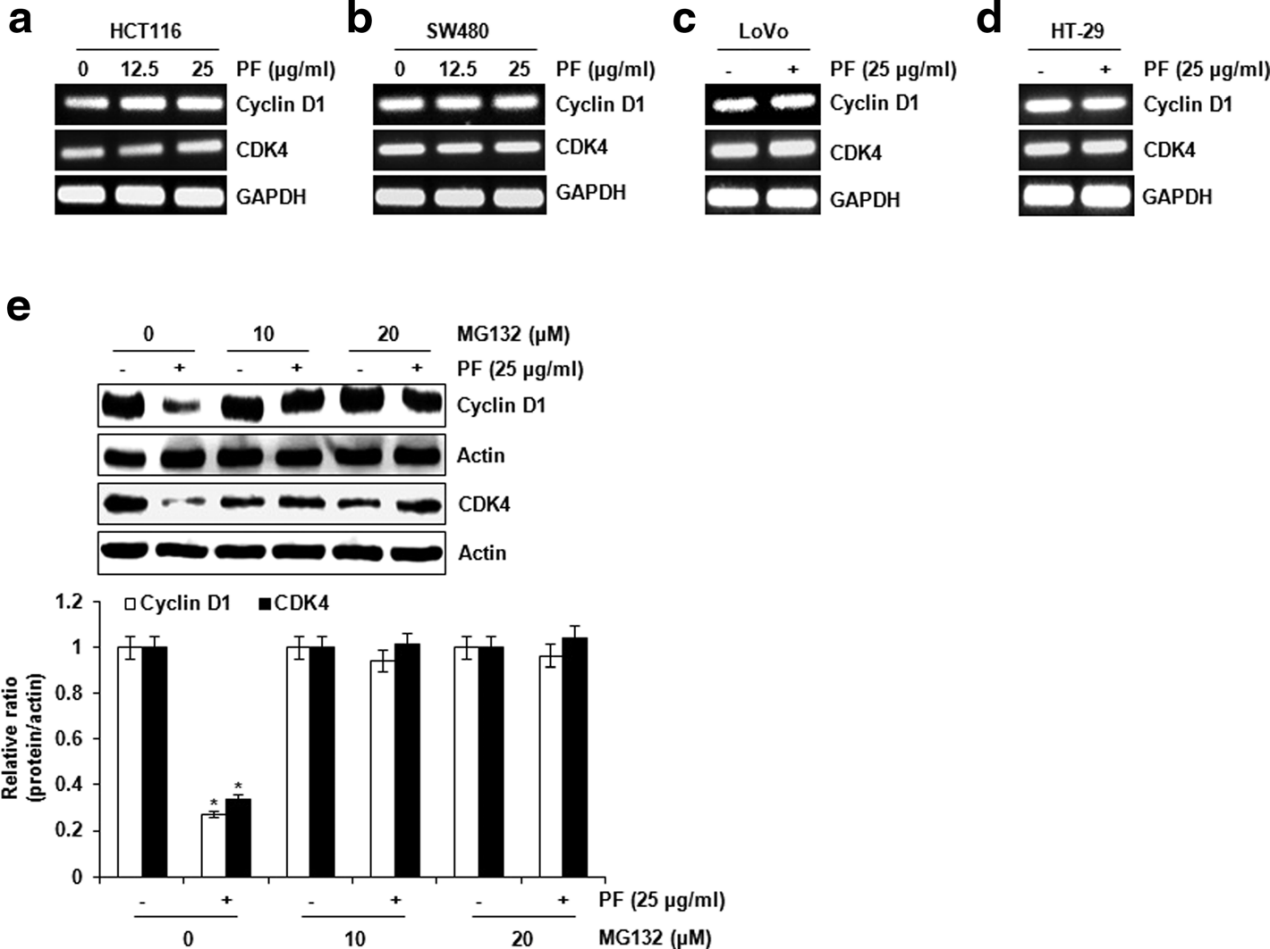
PF induces the proteasomal degradation of cyclin D1 and CDK4. **a**-**d** The cells were treated with the indicated concentrations of PF for 24 h. For RT-PCR analysis of the gene expression of cyclin D1 and CDK4, total RNA was prepared after PF treatment for 24 h. GAPDH was used as internal control for RP-PCR. **e** HCT116 cells were pretreated with MG132 for 2 h, and then co-treated with PF (25 μg/ml) for 3 h (cyclin D1) or 10 h (CDK4). Cell lysates were subjected to SDS-PAGE and the Western blot was performed using antibodies against cyclin D1 and CDK4. Actin was used as internal control for Western blot analysis. *P < 0.05 compared to cell without PF treatment

To elucidate the potential mechanism by which PF down-regulates the protein level of cyclin D1 and CDK4, the cells were treated with PF, and then mRNA levels of cyclin D1 and CDK4 were evaluated using RT-PCR. As shown in Fig. 3a-d, the mRNA levels of cyclin D1 and CDK4 were not significantly changed in the presence of PF. This result indicates that PF may regulate the protein level of cyclin D1 and CDK4 through proteasomal-dependent degradation. To investigate whether PF affects the modification of cyclin D1 and CDK4 protein, HCT116 cells were pretreated with MG132 for 2 h, and then co-treated with PF for 3 h for cyclin D1 or 10 h for CDK4. As shown in Fig. 3e, the degradation of cyclin D1 and CDK4 protein was restored in presence of the proteasomal inhibitor, MG132, which suggests that PF down-regulates cyclin D1 and CDK4 protein at the post-translational level via the proteasomal pathway.

### Cyclin D1 degradation by PF is dependent on threonine-286 (Thr-286) phosphorylation

**Fig. 4:**
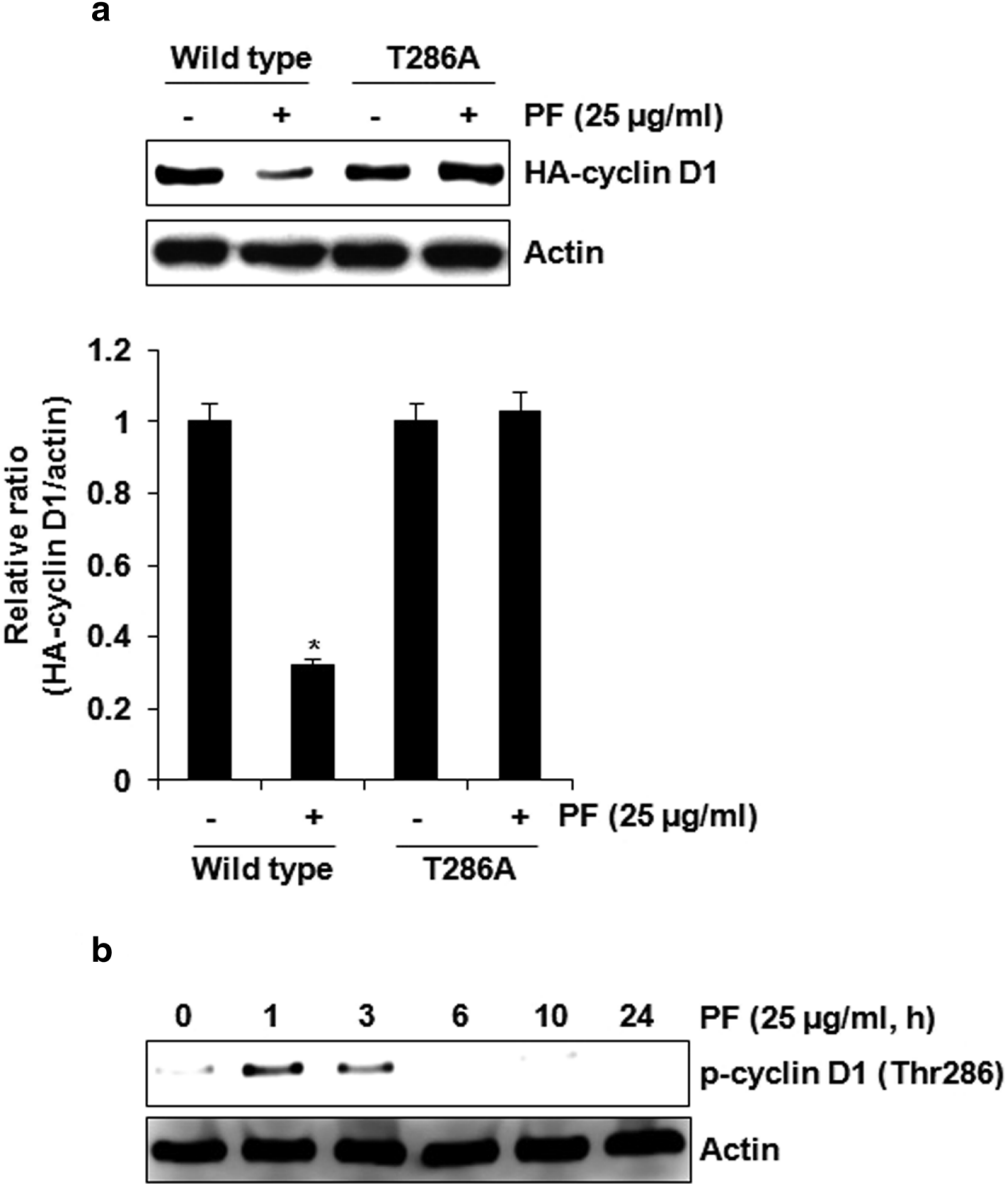
T286 phosphorylation of cyclin D1 by PF contributes to the proteasomal degradation. **a** HCT116 cells were transfected with wild type HA-tagged cyclin D1 or HA-tagged T286A cyclin D1 expression vector, and then treated with PF (25 μg/ml). *P < 0.05 compared to cell without PF treatment. **b** HCT116 cells were treated with PF (25 μg/ml) for the indicated times. Cell lysates were subjected to SDS-PAGE and the Western blot was performed using antibodies against HA-cyclin D1 and p-cyclin D1 (Thr286). Actin was used as internal control for Western blot analysis

Threonine-286 (Thr-286) phosphorylation of cyclin D and subsequent ubiquitination pathway has been reported to be the main cyclin D1 degradation pathway [15]. To investigate whether Thr-286 phosphorylation is critical in PF-mediated cyclin D1 degradation, HCT116 cells were transfected with HA-tagged wild type cyclin D1 and HA-tagged T286A cyclin D1. As shown in Fig. 4a, exogenous wild type cyclin D1 was decreased by PF, whereas the degradation of T286A cyclin D1 was suppressed. In addition, we observed that PF phosphorylated Thr-286 of cyclin D1 (Fig. 4b). These results suggest that Thr-286 site plays an important role in PF-induced cyclin D1 degradation.

### Upstream kinases associated PF-mediated degradation of cyclin D1 and CDK4 protein

**Fig. 5:**
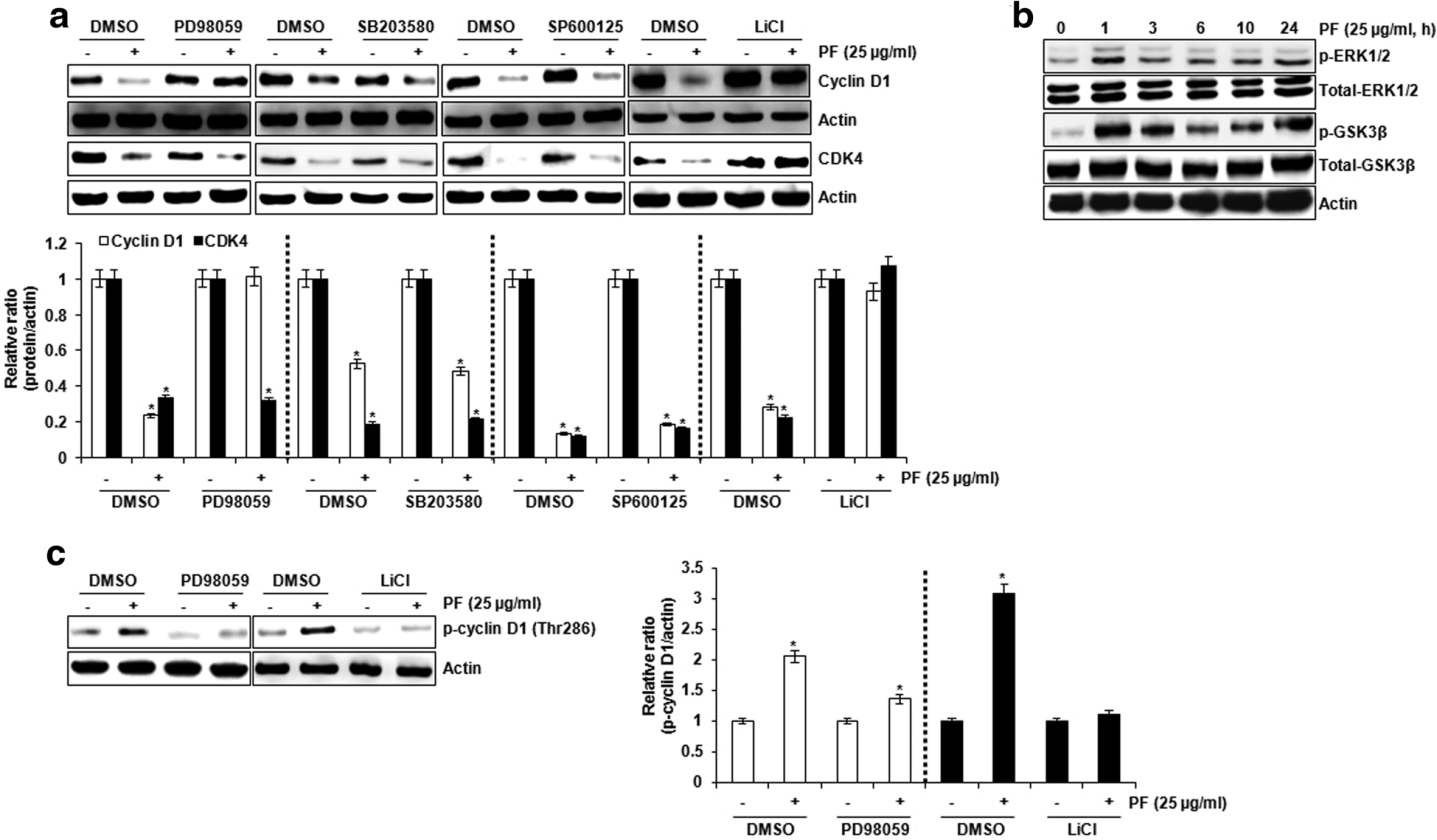
Determination of upstream kinases involved in the proteasomal degradation of cyclin D1 and CDK4 by PF. **a** HCT116 cells were pretreated with PD98059 (ERK1/2 inhibitor, 20 μM), SB203580 (p38 inhibitor, 20 μM), SP600125 (JNK inhibitor, 20 μM) or LiCl (GSK3β inhibitor, 20 mM) for 2 h, and then co-treated with PF (25 μg/ml) for 3 h (cyclin D1) or 10 h (CDK4). **b** HCT116 cells were treated with PF (25 μg/ml) for the indicated times. **c** HCT116 cells were pretreated with PD98059 (ERK1/2 inhibitor, 20 μM) or LiCl (GSK3β inhibitor, 20 mM) for 2 h, and then co-treated with PF (25 μg/ml) for 1 h. Cell lysates were subjected to SDS-PAGE and the Western blot was performed using antibodies against cyclin D1, CDK4, p-ERK1/2, total-ERK1/2, p-GSK3β, total-GSK3β or p-cyclin D1 (Thr286). Actin was used as internal control for Western blot analysis. *P < 0.05 compared to cell without PF treatment

To investigate the upstream kinases associated with PF-mediated degradation of cyclin D1 and CDK4 protein, HCT116 cells were pretreated with PD98059 (20 μM, inhibitor of extracellular signal–regulated kinase 1/2 (ERK1/2)), SB203580 (20 μM, p38 inhibitor), SP600125 (20 μM, inhibitor of c-Jun N-terminal kinases (JNK)) or LiCl (20 mM, inhibitor of glycogen synthase kinase 3β (GSK3β)) for 2 h, and then co-treated with PF for 3 h for cyclin D1 or 10 h for CDK4. As shown in Fig. 5a, cyclin D1 degradation by PF was attenuated in presence of the ERK1/2 and GSK3β inhibitor, whereas PF-mediated CDK4 degradation was blocked in presence of GSK3β inhibitor, which suggests that cyclin D1 degradation by PF is dependent on both ERK1/2 and GSK3β, and CDK4 degradation by PF is dependent on GSK3β. Thus, we examined whether PF induces the phosphorylation of these kinase as the active form. As shown in Fig. 5b, the phosphorylation of ERK1/2 and GSK3β was induced at early time points in the presence of PF. In addition, we observed the inhibition of ERK1/2 and GSK3β attenuated PF-mediated Thr-286 phosphorylation of cyclin D1 (Fig. 5c).

## Discussion

Psoralea Fructus (PF), the dried and ripe fruit of *Psoralea corylifolia* known as “*Bo-Gol-Zhee*” in Korea has been known as an important medicinal plant used in several traditional medicines to cure various diseases [3]. The extracts of PF have been reported to possess various pharmacological properties such as antibacterial, anticancer, antioxidant, anti-inflammatory, antifungal and immunomodulatory activity [3]. In anticancer activity, PF inhibited hypoxia-inducible factor-1 (HIF-1) activation in human gastric cancer cells [16]. Administration of ethanol extract of PF (100 and 200 mg/kg) inhibited EAC ascitic tumor growth [17]. In addition, PF has been reported to possess cytotoxic activity against human stomach carcinoma, colorectal cancer and breast cancer cells [18, 19] and to induce the cell growth arrest in human oral carcinoma lines and erythroleukemia cells [5].

Cyclin D1 and CDK4 have been regarded as cell cycle regulators that control the G1 to S phase of the cell cycle. These proteins have been considered pivotal target proteins in various cancers because many anti-cancer drugs can inhibit the expression of cyclin D1 and CDK4. Thus, the investigation of the molecular mechanism by which PF down-regulates cyclin D1 and CDK4 expression may be required to understand how to better treat cancer and even to develop better anti-cancer drugs. In this study, we showed that the protein levels of cyclin D1 and CDK4 is reduced but not mRNA levels in presence of PF, which indicates that PF may regulate the protein level of cyclin D1 and CDK4 through proteasomal-dependent degradation. Cyclin D1 and CDK4 have been known to be degraded at the translational level [8, 20]. For cyclin D1 proteasomal degradation, several modifications such as RXXL motif, T286 and lysine residues have been identified [8]. HCT116 cells treated PF and MG132 as the proteasome inhibitor exhibited no degradation of cyclin D1, and T286A blocked cyclin D1 degradation by PF. These findings suggest that the downregulation of cyclin D1 by PF may result from cyclin D1 proteasomal degradation via T286 phosphorylation. In addition, we observed that PF-mediated decrease of CDK4 protein was blocked in presence of MG132, showing that PF induces CDK4 proteasomal degradation. However, we did not determine how to induce CDK4 proteasomal degradation by PF. Indeed, C/EBPα has been reported to be involved in CDK4 proteasomal degradation [20]. CCAAT-enhancer-binding protein α (C/EBPα) overexpression leads to a reduction of CDK4 protein level, but not mRNA [20]. In addition, C/EBPα exerts the formation of CDK4-ubiquitin conjugates and induces CDK4 proteasomal degradation [20]. Thus, the study for the effect of PF on C/EBPα may be required to understand the mechanism for the induction of CDK4 proteasomal degradation by PF.

Cyclin D1 degradation dependent on T286 phosphorylation is associated with GSK3β activity [21]. In this study, GSK3β inhibition by LiCl attenuated PF-induced cyclin D1 degradation. In addition, we observed that the inhibition of ERK1/2 blocks cyclin D1 degradation by PF. Indeed, ERK1/2 has been reported to be involved in cyclin D1 proteasomal degradation [15, 22]. Although p38 and JNK is involved in cyclin D1 degradation [15, 22], these two kinases did not affect the downregulation of cyclin D1 by PF. These findings suggest that the upstream kinases involved in PF-mediated cyclin D1 degradation may be ERK1/2 and GSK3β. Interestingly, we found that the downregulation of CDK4 by PF is blocked by GSK3β inhibition by LiCl, indicating that the upstream kinase involved in PF-mediated CDK4 degradation may be GSK3β.

## Conclusions

This study supports the hypothesis that PF exerts anti-cancer activity, and downregulation of cyclin D1 and CDK4 plays a role in PF-induced anti-cancer activity. Our findings will provide the potential PF usage in the cancer drug development. Characterization of PF in in vivo could be required for the further.

## Abbreviations

C/EBPα: CCAAT-enhancer-binding protein α
CDK: Cyclin-dependent kinase
DMSO: Dimethyl sulfoxide
ERK1/2: Extracellular signal–regulated kinase 1/2
GSK3β: Glycogen synthase kinase 3β
JNK: c-Jun N-terminal kinases
MTT: 3-(4,5-dimethylthizaol-2-yl)-2,5-diphenyl tetrazolium bromide
PF: Psoralea Fructus
RT-PCR: Reverse transcriptase-polymerase chain reaction
Thr286: Threonine-286
